# Motoric performance variation from morning to evening: 80% intensity post-activation potentiation protocol impacts performance and its diurnal amplitude in basketball players

**DOI:** 10.3389/fpsyg.2022.1066026

**Published:** 2022-12-06

**Authors:** Özgür Eken, Elena Mainer-Pardos, Fatma Hilal Yagin, Ismihan Eken, Pablo Prieto-González, Hadi Nobari

**Affiliations:** ^1^Department of Physical Education and Sport Teaching, Inönü University, Malatya, Turkey; ^2^Health Sciences Faculty, Universidad San Jorge, Zaragoza, Spain; ^3^Department of Biostatistics and Medical Informatics, Faculty of Medicine, Inönü University, Malatya, Turkey; ^4^Department of Health and Physical Education, Prince Sultan University, Riyadh, Saudi Arabia; ^5^Faculty of Sport Sciences, University of Extremadura, Cáceres, Spain; ^6^Department of Motor Performance, Faculty of Physical Education and Mountain Sports, Transilvania University of Braşov, Braşov, Romania

**Keywords:** team sport, warm up, countermovement jump, PAP, seated medicine ball throw, diurnal variations

## Abstract

**Introduction:**

Post-activation potentiation (PAP) can be defined as the acute enhancement in muscle performance after preload stimulation that occurs during strength exercises or warm-up protocols, and the contractile history of any muscle group can directly influence the presented performance. The purpose of this study was to compare the results of motoric performance tests carried out at two different times of the day using three different PAP protocols.

**Methods:**

Thirty-two male basketball players with at least 2 years of training experience and who competed at the national level were recruited for this study [age: 21.80 ± 1.91 years; body height: 178.40 ± 6.85 cm; body mass: 72.50 ± 7.16 kg; body mass index (BMI): 22.81 ± 2.28]. After control and experimental-specific warm-up (SWU) sessions, participants completed a countermovement jump (CMJ) and seated medicine ball throw (SMBT). The SWU protocol was developed based on the athlete’s typical warm-up routine. The experimental warm-ups included the same exercises as the SWU, with one set of bench presses for five repetitions at 80% (80% PAP) and one repetition at 100% of 1 RM (100% PAP). Each of the protocols consists of 15 min. The SWU and experimental warm-up sessions were completed in a random, counterbalanced order, completed in a period of 3-weeks.

**Results:**

According to the research findings, carrying out the protocols in the evening led to a larger improvement in SMBT than in the morning (*F* = 35.94, *p* < 0.001, η*^2^_*p*_* = 0.537). Additionally, the SMBT results were statistically more significant in the group that received 80% PAP compared to the SWU (*p* = 0.034), and the SMBT results were also higher in the group that received 100% PAP compared to the SWU (*p* = 0.002). Furthermore, the interaction effect (time × PAP) was statistically significant in SMBT (*F* = 6.39, *p* = 0.003, η*^2^_*p*_* = 0.17).

**Discussion:**

These results may provide more particular recommendations than previously thought to basketball coaches for the planning of basketball-specific PAP exercises prior to the start of training programs.

## Introduction

Basketball is known for its intermittent high-intensity activities during four 10 min quarters, with a 15 min break between the second and third quarters and only a 2 min break between the first and fourth quarters (Basketball Rules, 2022). It requires developing physical, technical, tactical, and psychological qualities to succeed (Ziv and Lidor, 2009). For these reasons, warm-up strategies that ensure their optimal need to be developed (Stevanovic et al., 2019; Eken, 2021, 2022; Räisänen et al., 2021).

Resistance training or warm-up procedures can bring about a post-activation potentiation (PAP) phenomenon, defined as an immediate improvement in muscle performance brought on by preload stimulation (de Hoyo et al., 2015; Dello Iacono et al., 2016). Any muscle group’s contractile history can influence the performance that is demonstrated directly. The PAP refers to the physiological state that results in an abrupt increase in muscle strength and naturally occurring performance following preconditioning exercise (Chiu et al., 2003). Acute recovery can be brought about as a result of the mechanical, metabolic, and neuromuscular changes that occur during exercise. Nevertheless, the PAP mechanism causes an increase in the cross-bridge binding rate during muscle contraction. This is accomplished by the phosphorylation of myosin regulatory chains (de Hoyo et al., 2015; Bauer et al., 2019; Beato et al., 2021). This condition is brought on by increased sensitivity of proteins responsible for contractile activity to calcium produced by the sarcoplasmic reticulum (Baudry and Duchateau, 2007; Tillin and Bishop, 2009; Bauer et al., 2019).

It has been reported that the PAP effect starts approximately 3 min after the resistance exercise is performed before the sports-specific movements and the effect lasts for 10 min (Beato et al., 2019). However, there is no consensus regarding the onset of the effect (McBride et al., 2005). Many studies in the literature stated that a high-intensity preloading protocol should be applied to athletes to create the PAP effect (Lim and Kong, 2013; Seitz et al., 2014; Carbone et al., 2020). In this regard, resistance exercises, including isometric, concentric, or eccentric contractions, can be applied to achieve the PAP effect (Beato et al., 2021). Furthermore, the effects of warm-up can be limited by the time of day (Eken et al., 2022). In this regard, the time of day is essential because it is associated with the circadian rhythm and its biological and hormonal responses (Teo et al., 2011b). In addition, other factors like physical performance and sleep quality can operate as mediators or moderators. Nevertheless, it appears that the time of day is crucial for final physical and physiological performance in team sports (Thun et al., 2015). To date, it is evident that there are many elements to consider when attempting to perform PAP.

Studies examine the effect of PAP on team sports performance after resistance and overload exercises (Tillin and Bishop, 2009; Dello Iacono et al., 2018; Gepfert et al., 2020; Beato et al., 2021). Gepfert et al. (2020) researched the PAP effect in resistance training in 5 m slide-step movement performance in basketball players. The study revealed that a light resistance-assisted conditioning activity (5% of body mass) elicits a potentiation response in this population. Van Den Tillaar and Von Heimburg (2017) observed a significantly faster 20 m sprint time improvement after resisted sprints (absolute load of 5 kg for all participants) in handball players. In contrast, Till and Cooke (2009) evaluated the effect of resistance training consisting of dynamic and isometric exercises on soccer players’ sprint performance. There were no improvements in sprint times following resistance training; therefore, the results did not indicate a significant PAP impact (Till and Cooke, 2009).

Several studies examined the PAP effect of heavy resistance exercises performed in soccer (McBride et al., 2005; Till and Cooke, 2009; Zois et al., 2011; Low et al., 2015; Hammami et al., 2017; Sanchez-Sanchez et al., 2018). According to the literature, repetitive sprinting with heavy resistance exercises in soccer (Low et al., 2015; Sanchez-Sanchez et al., 2018), linear sprint (McBride et al., 2005; Till and Cooke, 2009), vertical jump, and change of direction skills (Zois et al., 2011) improved. Petisco et al. (2019) reported in their study with professional soccer players that moderate warm-up intensity can induce greater PAP, including improvements in jumping and repeated and non-repeatable deflection speed. Chatzopoulos et al. (2007) showed that resistance training with high load using a barbell squat with a load of 90% of maximal individual load enhanced 10 and 30 m sprint performance in team sports players, including basketball players. In a study examining the effects of Electro-myostimulation (EMS) as a PAP protocol on sports performance. Sari et al. (2022) aimed to create a more significant PAP effect with more intense contractions with the application of EMS to amateur soccer players and rugby players. The study found no significant difference in the participants’ 10 and 30 m sprint performances (Sari et al., 2022). In addition, it was stated that PAP responses were related to the activated muscle fiber type (Wilson et al., 2013). As the fast fiber ratio increases, there is an increase in the size ratio of PAP responses (Sole et al., 2013).

To the authors’ knowledge, although there are studies investigating the relationship between training type and different kinds of team sports in the literature, there is no study examining the relationship between PAP protocol and diurnal variations. Besides, more research is needed about the effects of different PAP protocols on basketball players’ performance while considering the time of the day as an essential factor in identifying such an effect. Research that provides such a design may help coaches identify the adequate scenario and PAP to positively influence basketball players’ readiness for competitions. Therefore, the aim of this study was two-fold: (1) to analyze the effects of different PAP protocols on the performance of male basketball players determined during some motoric performances and (2) to identify the possible interactions of PAP with the time of the day.

## Materials and methods

### Participants

Male basketball players [*n* = 32; age: 21.80 ± 1.91 years; body height: 178.40 ± 6.85 cm; body mass: 72.50 ± 7.16 kg; body mass index (BMI): 22.81 ± 2.28] with ≥2 years of training and national competition experience were recruited for the study. At least twice per week of resistance training had been a part of each athlete’s routine for at least a year. The study was carried out 14 days following the conclusion of the national championship. Before the beginning of the investigation, in-depth information regarding the study’s scope, purpose, and methodology was provided to the participants who volunteered their time. The participant voluntarily agreed to participate in the research and indicated their willingness to do so by signing an informed consent form. Also, all the research followed the established international guidelines for the ethical study of human biological rhythms (Portaluppi et al., 2010). Participants in the study were instructed to get at least 8 h of sleep each night before their scheduled testing sessions. They were also instructed to arrive with a full stomach, with the proviso that they eat something at least 2 h before the morning and evening sessions. All tests and assessments applied in this study were approved by the Institute’s Clinical Research Ethics Committee (Approval Number: 3150/2022). During the implementation and testing phases of the protocols, the participants were also informed about the significance of refraining from high-intensity exercise and avoiding substances such as alcohol and caffeine. This information was relayed to the participants during the phases of implementation and testing of the protocols (Reilly et al., 2007).

### Methodology

**FIGURE 1:**
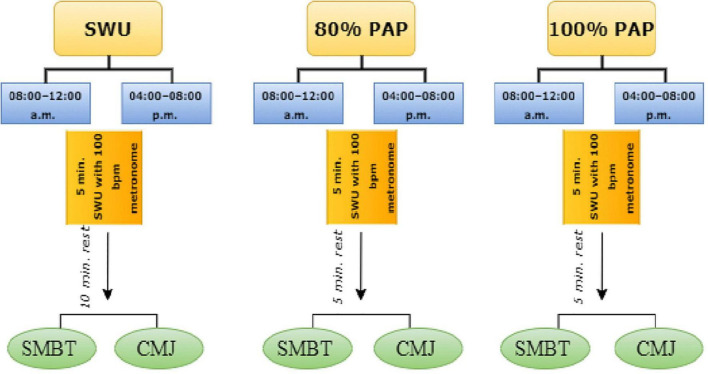
The flow chart for experimental design of research.

After the conclusion of the competitive period of the 2022 season, the trials were carried out. The tests during specific warm-up (SWU) and experimental sessions were completed in the same order, between 08:00 and 12:00 a.m. and 4.00 and 08.00 p.m., at an indoor sports center, with the same sports clothes and by the same investigator, who was blinded to the group allocation of the participants. To avoid the effects of fatigue on testing results, participants completed the SWU and the experimental warm-up sessions no less than 48 h after the last training/competition session. The countermovement jump (CMJ) and seated medicine ball throw (SMBT) performance of the participants were assessed after different PAP protocols, including; 5 min SWU with 100 bpm metronome and 10 min rest, 5 min SWU with 100 bpm metronome, 5 min rest and 80% PAP and 5 min SWU with 100 bpm metronome, 5 min rest and 100% PAP in two different time intervals within the same day, separated by at least 2 days in between each other. The amount of time needed to recover between the CMJ and the SMBT performance test was 1 min. Besides, all of the protocols consist of 15 min. All protocols were completed in a random, counterbalanced order, completed in a period of 3-weeks. All protocols continued for consecutive days. The testing procedures were carried out in the same locations the athletes often use for training and competition. In addition, in the protocols for obtaining PAP effects, the participants did bench presses for a maximum of five repetitions (5RM) and one repetition at maximum intensity (1RM) (Brzycki, 1993). This calculation was carried out during the control session, and in the experimental sessions, it was applied to determine 1RM and 5RM. The experimental design of the research is visually reported in the flowchart below (Figure 1).

#### Warm-up protocols

The overview of warm-up protocols is presented in Table 1. The SWU was designed according to the athlete’s regular warm-up practices. The SWU consisted of a 5-min specific warm-up with a 100 bpm metronome. The SWU exercise of the research is visually reported in Table 1. This SWU consisted of six different exercises (Boyle, 2004).

**TABLE 1:** Specific warm-up protocol.

SWU protocol	Description
Stationary Spider-Man	In the stationary Spider-Man, take a push-up position and attempt to step outside the right hand with the right foot.
Inchworm	Get into the push-up position to start. After getting into the push-up posture, lower the hips to stretch the abdominal region, and then walk the feet up as near the hands as he/she can while maintaining the straight position of the legs. This is accomplished by taking tiny steps without bending at the knees.
Backward and forward lunge walks	Backward and forward lunge walks are exercises used in warm-ups for the lower extremity, which involve all the leg and hip extensors while stretching out the anterior hip.
Backpedal	Another action involving moving backward is the backpedal, which targets the quadriceps rather than the hamstrings when it comes to warm-up.
Straight-leg skip	The skip with straight legs works the hip flexors while simultaneously increasing the dynamic strain on the hamstrings.
Heel-ups	The movement begins with an upright stance with flat feet on the ground. The participant’s weight should be evenly distributed between their feet, and their heels should be lifted to a comfortable height.

#### Anthropometric measurements

Body weights were determined using an electronic scale (Tanita SC-330S, Amsterdam, Netherlands) with an accuracy of 0.1 kg when measuring individual subjects (kg). During the measuring process, each participant’s height was determined using a stadiometer (Seca Ltd., Bonn, Germany) with an accuracy of 0.01 m. An electronic scale was used to measure and record each volunteer’s BMI as well as their ratio of lean to fat body mass (Tanita SC-330S, Amsterdam, Netherlands) (American College of Sports Medicine, 2018).

#### Countermovement jump

We evaluated the CMJ with a force platform from Kistler (Winterthur, Switzerland) at a sampling rate of 1,000 Hz (Meylan et al., 2010). The participants were instructed to stand and instantly leap after lowering themselves to self-selected knee flexion. They were also encouraged to perform each jump to their full potential. Under the watchful eye of a seasoned observer, the test volunteers were instructed to keep their hands on their hips and refrain from bending their knees before landing. Additionally, they were directed to avoid any knee flexion before landing. The best jump height (cm) result was recorded after the subjects completed two trials with 90 s of rest in between (Reeve and Tyler, 2013).

#### The seated medicine ball throw

This test is intended to evaluate the explosive strength of the upper body. The participant is seated with their back against the wall, legs completely extended, and feet apart. The athlete’s two hands are on a 2-kg medicine ball held in a position similar to the basketball passing stance. This competition aims to throw the ball as far as possible in a straight line. The distance is denoted in centimeters. The examination was taken thrice, and the score that represented the candidate’s performance the best was the one that was kept. In the course of the investigation, the dependability of the test was established. Every athlete was then given two trials to reduce their error, with a 90-s break in between (Van den Tillaar and Marques, 2013).

### Statistical analysis

A two-way repeated-measures analysis of variance (time–group interaction) was used in the study. The assumption of normal distribution was examined using the Shapiro–Wilk test. Mauchly’s test for sphericity was performed for the sphericity assumption. A Greenhouse–Geisser correction for sphericity was used where necessary. The first factor in the study consisted of three groups: SWU, 80% PAP, and 100% PAP. The second factor consisted of two measurements: morning and evening. Analyzes were made for SMBT and CMJ measurements.

Results are presented as mean ± standard deviation. In addition, a partial eta square (η*^2^_*p*_*) was calculated to measure the effect size of the variables in this study. Results for hypothesis testing are presented with the overall *F*-statistic of ANOVA, the corresponding *p*-value, and η*^2^_*p*_*. Finally, *p* < 0.05 was considered significant. Analyzes were performed using Python 3.9 and IBM SPSS Statistics for Windows version 26.0 (New York, NY; USA).

## Results

Table 2 contains descriptive statistics regarding the demographic data of the participants. Thirty-two participants between the ages of 19 and 26 with a mean age of 21.80 ± 1.91 were included in the study. The mean height, weight, BMI, 100% 1RM bench press, and 80% with five repetitions bench press 2 of the participants were 178.40 ± 6.85, 72.50 ± 7.16, 22.81 ± 2.28, 56.78 ± 10.10, 48.06 ± 8.37, respectively.

**TABLE 2:** Descriptive statistics on the demographic data of the participants.

Variable	Mean ± SD
Height (cm)	178.40 ± 6.85
Body mass (kg)	72.50 ± 7.16
Age (years)	21.78 ± 1.91
BMI (kg/m^2^)	22.81 ± 2.28
% 100 1RM bench press (kg)	56.78 ± 10.10
% 80 with five repeat bench press (kg)	48.06 ± 8.37

BMI, body mass index; Mean, mean values; SD, standard deviation.

Table 3 shows the changes in the SMBT parameter of the participants. According to the study’s data, there was a statistically significant difference between the measurements in terms of SMBT (*F* = 35.94, *p*_1_ < 0.001, η*^2^_*p*_* = 0.537). A statistically significant difference was found between the groups (SWU, 80% PAP, and 100% PAP) in terms of SMBT (*F* = 14.89, *p*_2_ < 0.001, η*^2^_*p*_* = 0.32). *Post-hoc* analyzes showed a statistically significant difference in SMBT value between the SWU – 80% PAP (*p*_3_ = 0.034), SWU – 100% PAP (*p*_3_ = 0.002), and 80% PAP – 100% PAP groups (*p*_3_ < 0.001). In addition, the time difference between the groups for SMBT value in the study was statistically significant (*F* = 6.39, *p* = 0.003, η*^2^_*p*_* = 0.17). As a result, the interaction effect was statistically significant.

**TABLE 3:** Comparison of the measured values of SMBT.

Groups	Mean ± SD	Between measurements	Between groups	Interaction
			
		*F*-value	*F*-value	
			
		*p*_1_ value	*p*_2_ value	
			
		η*^2^_*p*_*	η*^2^_*p*_*	
SWU–SMBT–morning	573.00 ± 47.37	*F* = 35.94 *p*_1_ < 0.001 η*^2^_*p*_* = 0.53	*F* = 14.89 *p*_2_ < 0.001 η*^2^_*p*_* = 0.32 SWU–80% PAP *p*_3_ = 0.034 SWU–100% PAP *p*_3_ = 0.002 80% PAP–100% PAP *p*_3_ < 0.001	*F* = 6.39 *p* = 0.003 η*^2^_*p*_* = 0.17
SWU–SMBT–evening	575.94 ± 44.96			
80% PAP–SMBT–morning	585.56 ± 50.24			
80% PAP–SMBT–evening	587.08 ± 46.05			
100% PAP–SMBT–morning	578.85 ± 48.17			
100% PAP–SMBT–evening	584.54 ± 48.53			

Mean, mean values; SD, standard deviation; *p*_1_ value, significance test result between measurements; η^2^*_p_*, partial eta squared; *p*_2_ value, significance test result between groups; *p*_3_ value, the results of the in-group comparison significance test; SWU, specific warm-up; SMBT, seated medicine ball throw; PAP, post-activation potentiation.

The changes in the CMJ parameter of the participants are presented in Table 4. According to the study’s data, there was a statistically significant difference between the measurements in terms of CMJ value (*F* = 19.38, *p*_1_ < 0.001, η*^2^_*p*_* = 0.384). A statistically significant difference was found between the groups (SWU, 80% PAP, and 100% PAP) in the study in terms of CMJ value (*F* = 8.07, *p*_2_ < 0.001, η*^2^_*p*_* = 0.206). *Post-hoc* analyses showed a statistically significant difference between the SWU and 80% PAP groups regarding CMJ value (*p*_3_ < 0.001). On the other hand, there was no statistically significant difference between SWU – 100% PAP and 80% PAP – 100% PAP groups in terms of CMJ value (*p*_3_ > 0.05). For the CMJ value, the difference between groups according to time was not statistically significant (*F* = 0.549, *p* = 0.536, η*^2^_*p*_* = 0.017). The interaction effect was not statistically significant (*p* > 0.05).

**TABLE 4:** Comparison of the measured values of CMJ.

Groups	Mean ± SD	Between measurements	Between groups	Interaction
			
		*F*-value	*F*-value	
			
		*p*_1_ value	*p*_2_ value	
			
		η*^2^_*p*_*	η*^2^_*p*_*	
SWU–CMJ–morning	42.30 ± 4.32	*F* = 19.38 *p*_1_ < 0.001 η*^2^_*p*_* = 0.38	*F* = 8.07 *p*_2_ < 0.001 η*^2^_*p*_* = 0.206 SWU–80% PAP *p*_3_ < 0.001 SWU–100% PAP *p*_3_ = 0.128 80% PAP–100% PAP *p*_3_ = 0.23	*F* = 0.549 *p* = 0.536 η*^2^_*p*_* = 0.017
SWU–CMJ–evening	43.94 ± 4.79			
80% PAP–CMJ–morning	44.09 ± 3.85			
80% PAP–CMJ–evening	45.50 ± 4.23			
100% PAP–CMJ–morning	43.47 ± 3.83			
100% PAP–CMJ–evening	44.75 ± 4.47			

Mean, mean values; SD, standard deviation; *p*_1_ value, significance test result between measurements; η^2^*_p_*, partial eta squared; *p*_2_ value, significance test result between groups; *p*_3_ value, the results of the in-group comparison significance test; SWU, specific warm-up; CMJ, countermovement jump; PAP, post-activation potentiation.

## Discussion

This study aimed to determine the differences in some motoric performance assisted at two different times of day (morning and evening) after three different PAP protocols (SWU, 80% PAP, and 100% PAP). The results obtained for CMJ measurements showed that performing the exercise in the evening increased the CMJ value compared to performing the exercise in the morning. According to the results obtained for the study groups, it can be said that the CMJ value is higher in the 80% PAP group than in the SWU group. On the contrary, there was no statistically significant difference between SWU and 100% PAP and 80% PAP and 100% PAP groups in terms of CMJ value. Also, time × group interaction was not significant for CMJ. The results revealed that evening exercise or 80% PAP positively affected CMJ measurements. In the past, improvements in jumping performance following loaded squat PAP procedures have been documented in male athletes competing in team sports such as rugby, volleyball, and soccer (Gouvêa et al., 2013). These athletes’ leaping performance improved after the protocol. These findings can be explained by several short-term neuromechanical adaptations, such as increased muscle-tendon stiffness (Till and Cooke, 2009). In addition, the effects of PAP are contingent upon the equilibrium between neuromuscular weakening and muscle weariness (Tillin and Bishop, 2009). According to the reports, the outcome of this circumstance is determined by the density following the load being utilized (Sale, 2004). Petisco et al. (2019) compared the effects of various intensities of warm-up conditioning on the physical fitness of male professional field soccer players. All of the jumps [a squat jump (SJ) and a CMJ] exhibited possible to probable enhancements. In addition, they examined that a change of direction ability (COD) test, a repeated sprint with a COD, performed after the 80%-1RM warm-up procedure was superior to the 100%-1RM and 60%-1RM warm-up protocols (Petisco et al., 2019). In the current study, a load of moderate intensity (i.e., 80% of 1RM) induced more significant jumping performance improvements than loads of greater intensity (i.e., 100% of 1RM). This finding is consistent with earlier research that found more significant PAP effects after loads of intermediate intensity in team sports (Gouvêa et al., 2013; Petisco et al., 2019). The results of the study are similar to our study. Physiologically similar results may be due to the following reasons. Saez Saez de Villarreal et al. (2007) evaluated the influence of several forms of active warm-up stimuli of muscle activation on explosive jumping performance after short (5 min postwarm-up) and long (6 h postwarm-up) recovery periods following the warm-up. The study showed that high-intensity dynamic loading (e.g., 80–95% 1RM) and the specialized volleyball warm-up approach had the largest effect on subsequent neuromuscular explosive responses. It has been determined that the positive effects of a warm-up on jump performance are maintained even after long recovery periods (e.g., 6 h after the warm-up), especially when high-intensity dynamic movements were performed before the warm-up (Saez Saez de Villarreal et al., 2007). The reason for this results may be the activation of fast twitch muscle fibers, which is considered a crucial element for activating PAP, resulting from PAP activities designed to optimize movement speed (Hamada et al., 2003). It is crucial to highlight that the increased PAP effect that occurs following intermediate-intensity loads may be particularly relevant when PAP activities are carried out to achieve the tremendous possible increase in movement velocity (Turner et al., 2015). Even after 6 h had passed since the PAP warm-up, it was possible to see an improvement in leaping performance following loaded squats (Sanchez-Sanchez et al., 2018). Some research may be found in the literature mentioned above, including basketball players. Gepfert et al. (2020) found a statistically significant difference in the performance levels of subjects before and after receiving post-assisted PAP. They found no significant intragroup differences between preconditioning and post-conditioning activities (Gepfert et al., 2020). Similar to the results in our study, PAP protocols led to improved performance results. Pożarowszczyk et al. (2018) investigated the effects of PAP on the thickness, elasticity, and stiffness of the Achilles tendon (AT) in basketball players. After physical exertion, the stiffness of the AT rose dramatically relative to its initial value, although its thickness and elasticity decreased. The PAP exercise significantly altered the AT’s rigidity, flexibility, and thickness (Pożarowszczyk et al., 2018). Although the results of this study conclude that the PAP effect has a positive effect, the measured parameters differ. Dello Iacono et al. (2020) examined the impact of two PAP protocols utilizing traditional-set or cluster-set configurations on the execution of CMJ. They showed that both treatments induced PAP responses in vertical jump performance when jump squats were performed at the optimal power load. Nonetheless, the cluster-set design resulted in higher performance at all time points, presumably due to lower muscular exhaustion (Dello Iacono et al., 2020). Similar to the findings of our investigation, it was found in this study that PAP protocols impacted the performance values. Pociūnas et al. (2018) examined the acute effects of various half-time re-warm ups on vertical jump height in simulated basketball games. All three methods of halftime re-warm ups (aerobic + post-activation potentiation exercises and aerobic + post-activation potentiation + stabilizing activities) resulted in a non-significant decrease in the CMJ height during simulated basketball games (Pociūnas et al., 2018). The mechanisms underlying the power reduction hypothesis may relate to tactical aspects of the game, such as fatigue, halftime length, and temperature (Mugglestone et al., 2012; Zois et al., 2013).

The results of our study indicate that nighttime exercise increases SMBT compared to morning exercise. There was a statistically significant difference in SMBT outcomes between the SWU, 80% PAP, and 100% PAP groups. The SMBT value in the 80% PAP group was significantly greater than in the SWU and 100% PAP groups. In addition, a statistically significant improvement in SMBT scores was seen between the 100% PAP group and the SWU group. From this perspective, we can conclude that PAP has a favorable effect on the SMBT value of athletes and that the PAP degree (%) affects the SMBT value of athletes. In addition, the effect of group × time interaction on the SMBT value was considerable.

The interaction effect results indicated that performing SMBT in the evening with 80% PAP will improve results. Mensur et al. (2018) found the ideal rest interval between PAP and medicine ball tossing and confirmed that the performance of the explosive strength of the upper limbs was enhanced when it was preceded by 3 × 3 90% of 1RM on the inclined bench press. They concluded that 3 × 3-90% 1RM incline bench press (IBP) results in a statistically significant increase in the distance of the medical ball throw from the chest and that 7 min is the optimal recovery time after an activation stimulus for amateurs (Mensur et al., 2018). Faigenbaum et al. (2006) studied the acute effects of four warm-up procedures with and without a weighted vest on high school female athletes’ anaerobic performance. They reported no statistically significant changes between trials for the sitting medicine ball throw or 10-yard sprint. These results may be since although some of my lower body dynamic jump moves require strong arm movement, only vigorous push-ups are practiced with a particular focus on the upper body. Therefore, it has been reported that measures of upper body strength performance, reaction time, and sprint speed are less likely to be positively or negatively affected by the design of the warm-up protocol (Faigenbaum et al., 2006). In our study, however, no different warm-up activation was performed and our results differed according to this study. Kalmus et al. (2021) investigated the short-term effects of two equal low-volume resistance training protocols, velocity-controlled (VC) and repetition to failure (RTF), on upper and lower body performance in competitive male adolescent basketball players. Both the CMJ and MBT increased 6 and 24 h after VC and RTF, respectively. After 24 h, only VC enhanced CMJ (Kalmus et al., 2021). This study examined the performance enhancement following activation at 6 and 24 h, during which the calcium sensitivity generated by greater myosin light chain phosphorylation would diffuse (Vandenboom et al., 1993). However, it has been postulated that PAPE may be caused by increased synaptic activity between afferent terminals and α-motoneurons, as well as other mechanisms linked with a delayed potentiation effect, including neurological variables, body temperature, and water content (Prieske et al., 2020).

Several studies have investigated the effect of time of day on basketball players’ performance (Heishman et al., 2017). Heishman et al. (2017) examined the difference in performance, player preparation, and self-reported sleep between morning and afternoon training sessions for male collegiate basketball players. According to the study, morning training reduces performance and reduces self-reported sleep duration (Heishman et al., 2017). Although the purpose of this study was not to investigate the mechanism by which sleep duration influences performance, it is possible that daily fluctuations in hormone levels play a role (Sedliak et al., 2007; Teo et al., 2011a; Smith et al., 2013). In light of the literature, it has been shown that cortisol levels may rise after awakening (Schmidt-Reinwald et al., 1999), and that morning salivary cortisol levels are connected with performance declines (Sinclair et al., 2013). Although no evaluation was made regarding the preparation and self-reported sleep of basketball players in our study, the fact that it had a positive effect on performance values measured in the evening is similar to our study. Pengelly et al. (2022) investigated chronotype-dependent diurnal variations in basketball shooting accuracy. The researchers examined non-significant changes in shooting scores between morning and afternoon trials for each chronotype group, with small-to-large impacts favoring the morning across all chronotypes.

Furthermore, variations in shooting scores between chronotype groups in the morning (small-large effects) and afternoon were not statistically significant (moderate-large effects) (Pengelly et al., 2022). Pengelly et al. (2021) identified the effect of player chronotype on in-game basketball performance during evening games. The study found that in-game performance appeared not to be affected by chronotype in evening games among professional male basketball players (Pengelly et al., 2021). Gepfert et al. (2020) investigated whether 5% body mass-resisted or assisted conditioning activity (CA) may improve 5 m slide-step performance. They had found a statistically significant difference between baseline performance and post-assisted postactivation performance increase (PAPE; Gepfert et al., 2020). The intensity with which the CA was implemented may be responsible for the discrepancies between their study’s findings and those of earlier research. Taking into account the athlete’s strength level, a higher intensity of the CA may be required to elicit the potentiation effect (Mero and Komi, 1986).

The findings of this investigation are limited in some ways. During this research, the afternoon hours were not investigated, nor were any subjects other than male basketball players considered. Expanding the pool of participants to include more female basketball players of varying ages makes it easier to replicate the study. In addition, it can provide more specific recommendations for increasing the number of studies examining the effects of different PAP protocols and diurnal variations on various performance parameters in basketball players, as well as for planning different PAP protocols for basketball players before training programs.

## Conclusion

In conclusion, the current investigation findings demonstrate that the time of day and the PAP substantially influence SMBT and CMJ. The application that imitates basketball and is closely related to the distinctive basketball framework is favored for use with the PAP. It was reported that the PAP effect occurring in the evening hours improved the performance of SMBT and CMJ. The results of this study can provide valuable insight into the design of training programs. Basketball players may find that increasing their performance due to this factor is possible.

## Data availability statement

The datasets presented in this article are not readily available because in accordance with the rules of the Ethics Committee that approved the study to protect the identity of the participants. Requests to access the datasets should be directed to HN.

## Ethics statement

The studies involving human participants were reviewed and approved by the Institute’s Clinical Research Ethics Committee. The patients/participants provided their written informed consent to participate in this study.

## Author contributions

ÖE, EM-P, FHY, IE, and HN: conceptualization and data curation. ÖE, FHY, and IE: formal analysis. PP-G: funding acquisition. ÖE: methodology. ÖE, EM-P, FHY, PP-G, and HN: supervision. ÖE, EM-P, FHY, IE, PP-G, and HN: writing—original draft and writing—review and editing. All authors contributed to the article and approved the submitted version.
